# Youth drinking in decline: What are the implications for public health, public policy and public debate?

**DOI:** 10.1016/j.drugpo.2022.103606

**Published:** 2022-04

**Authors:** John Holmes, Hannah Fairbrother, Michael Livingston, Petra Sylvia Meier, Melissa Oldham, Amy Pennay, Victoria Whitaker

**Affiliations:** a School of Health and Related Research, University of Sheffield, 30 Regent Street, Sheffield, S1 4DA, UK; b Health Sciences School, University of Sheffield, Barber House, 387 Glossop Road, Sheffield, S10 2HQ, UK; c National Drug Research Institute and enAble Institute, Faculty of Health Sciences, Curtin University, Perth, WA 6008, Australia; d Centre for Alcohol Policy Research, La Trobe University, Bundoora, Victoria, 3803, Australia; e MRC/CSO Social and Public Health Sciences Unit, University of Glasgow, 99 Berkeley Square, Glasgow, G3 7HR, UK; f Department of Behavioural Science and Health, University College London, 1-19 Torrington Place, London, WC1E 7HB, UK

**Keywords:** Alcohol, Trends, Young people, Policy process, Policy debate

## Abstract

Youth drinking has declined across most high-income countries in the last 20 years. Although researchers and commentators have explored the nature and drivers of decline, they have paid less attention to its implications. This matters because of the potential impact on contemporary and future public health, as well as on alcohol policy-making. This commentary therefore considers how youth drinking trends may develop in future, what this would mean for public health, and what it might mean for alcohol policy and debate.

We argue that the decline in youth drinking is well-established and unlikely to reverse, despite smaller declines and stabilising trends in recent years. Young people also appear to be carrying their lighter drinking into adulthood in at least some countries. This suggests we should expect large short- and long-term public health benefits. The latter may however be obscured in population-level data by increased harm arising from earlier, heavier drinking generations moving through the highest risk points in the life course.

The likely impact of the decline in youth drinking on public and policy debate is less clear. We explore the possibilities using two model scenarios, the reinforcement and withdrawal models. In the reinforcement model, a ‘virtuous’ circle of falling alcohol consumption, increasing public support for alcohol control policies and apparent policy successes facilitates progressive strengthening of policy, akin to that seen in the tobacco experience. In the withdrawal model, policy-makers turn their attention to other problems, public health advocates struggle to justify proposed interventions and existing policies erode over time as industry actors reassert and strengthen their partnerships with government around alcohol policy. We argue that disconnects between the tobacco experience and the reinforcement model make the withdrawal model a more plausible scenario. We conclude by suggesting some tentative ways forward for public health actors working in this space.

## Highlights

• The implications of recent declines in youth drinking require attention.
• Structural and cultural forces mean the declines are unlikely to be transient.
• Older heavy drinking cohorts mean harm rates may get worse before getting better.
• Alcohol may fall down the policy agenda leading to few new control policies.
• Stakeholders should be aware of the risks associated with reduced focus on alcohol.

## Introduction

Youth drinking has declined markedly across most high-income countries from the mid-2000s onwards. Compared to previous decades, young people are now less likely to drink alcohol and, if they do so, they start drinking at older ages, drink less often, consume smaller amounts and are less likely to get drunk (Kraus et al., 2018). The trends vary in scale and timing across countries (Vashishtha, Pennay, et al., 2020), but appear in both of the main international surveys detailing youth alcohol use; the Health Behaviours in School-aged Children study (HBSC, 2018, Fig. 1) and the European Schools Project on Alcohol and other Drugs (ESPAD) (Inchley et al., 2018; The ESPAD Group, 2020).

**Fig. 1:**
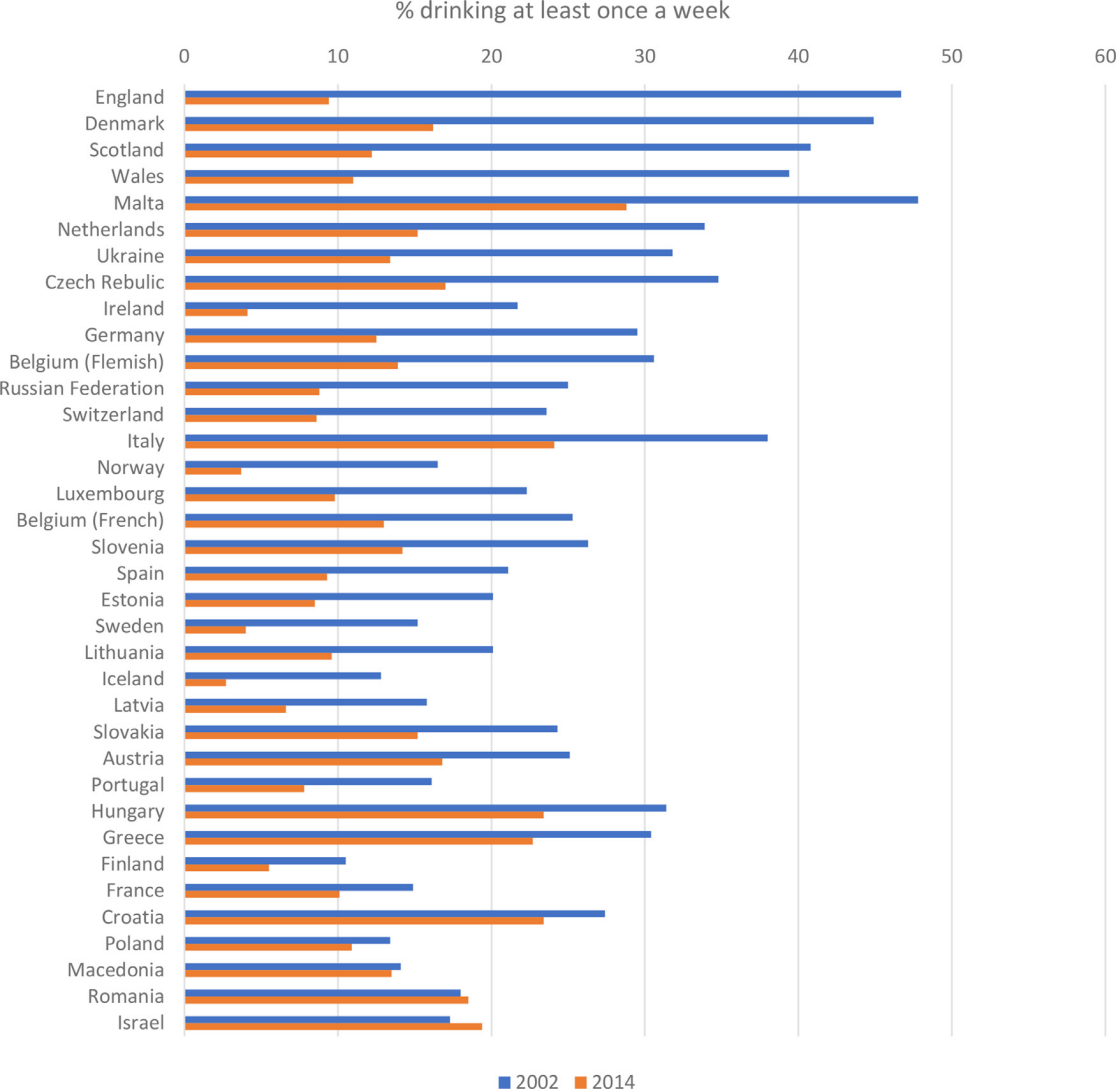
Prevalence of drinking at least once a week among 11-15 year-olds in the Health Behaviour in School-aged Children study. The figure sorts countries from largest to smallest percentage point change between 2002 and 2014. Iceland, Luxembourg, Slovakia and Romania did not provide data in 2002 and so this data point is for 2006 instead.

The decline in youth drinking has attracted substantial attention from researchers and commentators, who have characterised contemporary youth as ‘generation sensible’ and the ‘the new puritans’ (BBC News, 2018b; Financial Times, 2015). This contrasts starkly with discourse in the 1990s and 2000s that identified a ‘new culture of intoxication’ and ‘determined drunkenness’ among young people (Measham & Brain, 2005). However, while scientific and public debate have considered the nature and drivers of the decline, they have given much less attention to its implications, which may be profound.

Alcohol consumption is the leading risk factor globally for mortality and morbidity among 15-24 year-olds (Griswold et al., 2018), and is associated with a wide range of harmful outcomes. These include road traffic accidents, violence, poor educational performance, mental health problems and alcohol-specific conditions, such as alcohol poisoning and dependence (Bonnie & O'Connell, 2004). Initiation of drinking at younger ages and levels of drinking during young adulthood may also shape future public health by influencing alcohol consumption, and thus alcohol-related chronic disease, in later life (McCambridge et al., 2011; Meng et al., 2013). Drinking trends also matter for government and other policy stakeholders, including the public, as they influence the terms and tenor of public debate around young people and alcohol, while shaping judgements on the appropriateness, feasibility and design of alcohol policy options.

The COVID-19 pandemic raises further questions given widely reported changes to alcohol consumption patterns and practices among different population groups during periods of ‘lockdown’ restrictions (Acuff et al., 2021). There is little evidence specifically on changes in drinking among young people and it is therefore unclear whether understandings of the decline in youth drinking may shift as a ‘new normal’ and post-pandemic trends in alcohol consumption emerge.

This commentary paper examines what the decline in youth drinking means for current and future public health, as well as the implications for the arguments public health advocates make, the policies they recommend and the decisions taken by policy-makers, particularly politicians and civil servants. We structure our discussion around three questions. First, we ask how youth drinking trends will develop in future, as this has significant implications for the later questions. Second, we ask what the decline in youth drinking means for public health. Third, we ask what the decline means for public policy and debate and use two illustrative scenarios to show the range of possibilities. We then make recommendations on how public health actors can respond to the decline in youth drinking.

## How will youth drinking trends develop in future?

Evidence to date suggests the downturn in youth drinking may stabilise in the near future but is unlikely to reverse in the short-term, although the disruptive impact of the COVID-19 pandemic increases uncertainty on this point. The decline was steepest in the mid-2000s, as a wider group of Western European and Australian countries joined a trend that initially began in the US and Northern Europe across the late 1990s and early 2000s (Vashishtha, Pennay, et al., 2020). The most recent HBSC and ESPAD data from 2018-2019 suggest the decline is now slowing as trends stabilise in some countries, however there is little sign as yet of trends reversing or the downturn ending across all countries (Inchley et al., 2020; The ESPAD Group, 2020).

The international and long-term nature of the trend, and its consistency across population subgroups, also suggest it is unlikely to be transient, as these characteristics imply it is driven by largescale, long-term structural and cultural shifts (Room et al., 2020). These shifts may include increased economic insecurity among young people, the influence of new internet-based technologies, widespread shifts in relationships between parents and children, immigration from countries with more abstemious drinking cultures, and new health practices tied to notions of wellness and healthism (Pape et al., 2018; Rogne et al., 2019; Room et al., 2020; Törrönen et al., 2019; Vashishtha, Livingston, et al., 2020). Concurrent trends showing major shifts in a wide range of adolescent behaviours support this perspective. For example, Twenge's summary of evidence from US studies suggests young people are less likely than their predecessors to socialise without their parents, have sex, learn to drive, have a paid job or engage in extracurricular activities, such as sport or music (Twenge & Park, 2019). These interrelated shifts in the structures and practices of young people's everyday lives, and the demographic composition of the population, seem likely to hinder any rapid resurgence in youth drinking.

There is however significant debate regarding whether the decline in youth drinking will lead to a decline in adult drinking as today's young people move through the life course. Some researchers suggest young people may be delaying their initiation of alcohol consumption, rather than rejecting it entirely. This theory aligns with wider changes in young people's approach to adult responsibilities and pleasures (Caluzzi & Pennay, 2019; Twenge & Park, 2019). Twenge argues that the broad shifts she describes reflect a reduced desire among adolescents for freedom and a greater emphasis on spending time ‘cultivating the individual self’ (Twenge, 2017: p.42). This echoes evidence from qualitative studies that the cultural position of drinking has shifted among young people and it no longer represents a universal rite of passage (Törrönen et al., 2019). However, as young people reach adulthood, such considerations may dissipate and the drivers of drinking across recent decades will remain. These include high availability and affordability of off-trade alcohol in particular, pervasive marketing, gender norms that promote drinking among women and men, life stressors and the gastronomic and social pleasures associated with alcohol (Babor et al., 2010; Emslie et al., 2015; Kuntsche et al., 2006). The US provides a precedent for such a transition, with Keyes et al. finding that women born in the late 1970s and early 1980s consumed more alcohol as adults than both preceding and succeeding generations despite drinking less as adolescents (Keyes et al., 2019).

Current evidence however points towards the decline in youth drinking persisting into adulthood in some but not all countries (Dunphy, 2021). For example, the proportion of 16-24 year-olds in England who reported drinking in the last week fell from 67% in 2002 to 41% in 2019, making them the lightest drinking age group among adults (NHS Digital, 2020). Similarly, evidence comparing age trajectories of drinking for recent Australian birth cohorts suggest that the difference in drinking behaviours between cohorts narrows but does not close as they move through early adulthood (Livingston et al., 2021). In contrast, a Finnish study found differences in drinking between recent birth cohorts had disappeared by age 18 (Lintonen et al., 2016).

We broadly offer a positive view of whether the decline in youth drinking will persist, but some longer-term threats remain. The greatest of these may be complacency from policy actors and concerted action by alcohol producers. The decline may prompt erosion of the value of alcohol taxes, relaxation of advertising restrictions, reduced efforts by social responsibility organisations to curb the alcohol industry's worst excesses and less public discussion of alcohol-related health concerns. Industry actors may also shift from their current strategy of prioritising sales of higher priced, higher quality products (Pettigrew et al., 2018), and seek instead to increase sales volumes. Some researchers argue this attempt to regrow the overall alcohol market is already taking place through the development of new products (e.g. no- and low-alcohol drinks that may recruit young people to drinking), the use of marketing to link alcohol to leisure practices where it previously had no role (e.g. fitness and exercise) and the use of sophisticated social media strategies that speak to contemporary youth sensibilities by emphasising products’ authenticity and potential for creating ‘experiences’ (Curtis et al., 2018; Keric & Stafford, 2019).

Overall however, there remain reasons for optimism about the long-term prospects for the decline in youth drinking, even though the largest reductions may already have occurred. The more pressing question now appears to be whether and how these reductions will translate into improvements in public health.

## What does the decline in youth drinking mean for public health?

There are likely to be large public health benefits from a sustained reduction in youth drinking that persists into adulthood. In the short-term, rates of road traffic accidents, violence, alcohol poisonings and alcohol dependence should all decline among young people. The impact on alcohol-attributable diseases may be complicated however by long latency periods and competing trends. Alcohol-attributable mortality and morbidity from chronic disease peaks between ages 45 and 65, so we may not see the largest health benefits for some 30-40 years (Griswold et al., 2018). Indeed, while rates of harm may drop in the cohort of interest, overall rates of alcohol-related harm in some countries may get worse before they get better as the heavier drinking cohorts that attracted great concern during the 1990s and early 2000s move through the peak ages for alcohol-attributable harm (Meng et al., 2013). These benefits are of course contingent on the lower levels of drinking among today's adolescents persisting into adulthood. If this does not happen, any reduction in alcohol-related mortality and morbidity would be markedly smaller and concentrated on those acute conditions associated with younger age groups. Similarly, if the decline in youth drinking reverses, any reduction in alcohol-related harm would be smaller and more transitory.

To date, there is only limited published evidence on the impact of the decline in youth drinking on rates of alcohol-related harm. However, hospitalisation and mortality data from England indicates the scale of change that may occur in the long-term. For example, the rate of hospital admissions for alcohol-specific conditions (i.e. those attributable wholly to alcohol) among under-18s in England dropped by 58% from 72.1 to 30.7 per 100,000 between 2006/7-2008/9 and 2017/8-2019/20 (Public Health England, 2021). Similarly, the alcohol-specific mortality rate among 30-34 year-olds decreased by 22% between 2001 and 2019, despite large increases in older age groups (data for those aged under 30 are not publicly available) (Office for National Statistics, 2021). Comparable data from Australia is more equivocal however, with hospitalisation rates among 15-34 year-olds for conditions wholly attributable to alcohol broadly stable from 2012 to 2017 (National Drug Research Institute & Canadian Institute for Substance Use Research, 2021).

Three further points merit consideration when assessing the public health impact of the decline. First, the scale of impact also depends on trends in other health risk factors, such as smoking, illicit drug use and obesity. These risk factors often co-occur with alcohol use at the individual-level and interact with alcohol to magnify its health impacts (Taylor & Rehm, 2006; Whitaker et al., 2021). Many of these competing risk factors are also showing pronounced time trends among young people, with some risks becoming more prevalent (e.g. obesity, mental health problems) and others less prevalent (e.g. smoking and illicit drug use) (Patalay & Gage, 2019). Depending on the pattern of trends and interactions, public health benefits may be magnified or dampened. Second, *which* young people are reducing their alcohol consumption will also determine public health outcomes, as risks of harm are moderated by sociodemographic characteristics. In particular, the risk from higher levels of alcohol consumption is markedly greater for women and those in disadvantaged groups (Boyd et al.; Holmes et al., 2016). Studies to date suggest the downturn in drinking is present across the youth population, but may be smaller among girls, those of lower socioeconomic position and people who drink heavily (Pape et al., 2018), although evidence is mixed for the last two groups (Hallgren et al., 2012; Livingston, 2014; Oldham et al., 2020; Oldham et al., 2021; Raninen et al., 2013; Raninen et al., 2014; Zeebari et al., 2017). Third, a decline in alcohol-related harm concentrated within this lighter drinking generation would rebalance the age distribution of harm away from younger people and further concentrate harms among those in middle- and older-age. As we discuss below, this could have important implications for public debate and policy.

## What will the decline in youth drinking mean for public debate and policy?

Public policy and debate are shaped by a complex system of influences rather than simple linear processes of cause and effect. These influences include competing policy actors and networks, political considerations, power relations, formal and informal evidence processes, the organisational structures of policy-making institutions and structural forces such as economic and technological trends (Cairney, 2012). As such, it is unlikely that the decline in youth drinking will translate straightforwardly into simple and predictable changes in policy or debate. Given this, we do not make firm predictions but instead discuss two illustrative model scenarios, which we describe as the reinforcement and withdrawal scenarios and describe in detail below. We then consider additional potential features of alcohol policy debate that may arise from the decline in youth drinking.

### The reinforcement scenario

In the reinforcement scenario, an increasing number of adults abstain from alcohol consumption or drink moderately as lighter drinking generations move through the life course. Future generations also continue to drink less as they experience greater exposure to alternative health-related practices (e.g. mindfulness) via new technologies, rather than the concentrated alcohol marketing of broadcast media. Similarly, lower levels of parental drinking and closer parental relationships support still lighter drinking in adolescence. This creates favourable conditions for a shift in power within alcohol policy debate towards alcohol control advocates, as the general public, media organisations and key policy actors are less invested in opposing alcohol control policies (Gornall, 2014; Mercille, 2017). Steep reductions in alcohol consumption and related harm would also frame alcohol as a tractable problem, with policy options available and political rewards on offer for policy-makers willing to take effective action (Katikireddi et al., 2014). As such, the priorities and perspectives of public health advocates may align better with political incentives than those of industry actors.

Although industry-led arguments regarding the impact of alcohol control policies on people who drink lightly may still resonate, the shifts outlined above would still support moves away from the currently commonplace model of governments working in partnership with industry and towards greater partnership between government and public health actors. In turn, this would increase the likelihood that governments introduce stronger and more evidence-based alcohol control policies. Where governments are less willing to act, the international nature of the decline means external pressures may also force their hand. Such governments may be subject to processes of coercive policy transfer if, for example, supra-national organisations (e.g. the EU or WHO) adopt policies that national governments must follow or if, in a more likely situation, the adoption of policies by multiple governments increases the pressure on others to follow suit for fear of being out of step with international norms (Dolowitz & Marsh, 2000).

The reinforcement model partially replicates the tobacco experience, where a mutually reinforcing combination of sustained reductions in tobacco use, more negative public attitudes towards smoking, the accumulation of policies judged to be effective, and shifts within policy networks that gave increased influence to tobacco control advocates created a sustained and accelerating trend towards increased tobacco control (Nathanson, 1999; Studlar & Cairney, 2014). However, there are important differences between the tobacco experience and the current alcohol debate. First, there is little evidence that policy changes precipitated or sustained the decline in youth drinking (Bhattacharya, 2016; Vashishtha et al., 2020), and those that could be implicated are often associated with the industry-partnership approach to policy (e.g. the industry-backed Challenge 25 scheme to prevent underage sales in the UK). Second, while concerns about the health of smokers and those around them influenced tobacco trends and transformed tobacco policy debate and advocacy (Nathanson, 1999), there is much less evidence that specific health concerns about alcohol are a major driver of recent youth drinking trends. Indeed, the individual-level risk to most people who drink alcohol and those around them is not comparable in scale to that from smoking, even if the population-level harm may be (Holmes et al., 2016). Finally, smoking is typically framed as a binary behaviour within public and policy debate while alcohol consumption is framed as a continuum of consumption levels and practices, limiting the scope for a clear consensus ‘against’ drinking to emerge. The lack of public health-led policy successes specific to youth drinking, an individual-level health impetus or clear dividing lines that push advocates to opposite sides of the policy debate present important challenges for the reinforcement model. As a result, calls for new interventions may not resonate with the public, alcohol policy may not be a political priority and governments may only make incremental rather than radical changes.

### The withdrawal scenario

Given these points, we believe a withdrawal model is more likely. This envisages a more abstemious public who are apathetic towards rather than motivated to solve alcohol-related problems, which they are unlikely to experience and only then in the distant future (Lovatt et al., 2015). It also envisages an alcohol industry that harnesses or neutralises key drivers of the decline in youth drinking (e.g. by developing social media marketing that effectively counteracts competing messages). Policy-makers would also have fewer motivations to address alcohol-related harm, In some countries, there may also be increased concerns regarding the economic and cultural consequences of intervention, particularly in relation to traditional pubs or bars, and the wider night-time economy, which has experienced acute financial shocks during the COVID-19 pandemic. Consequently, public health advocates may struggle to attract government attention, partnership working between industry and government would continue and these partnerships would serve to remove or dilute restrictive policies as those involved argue such measures are no longer required or are actively harming economic and cultural assets (Miller & Harkins, 2010). In countries that have traditions or recent histories of implementing well-evidenced alcohol control policies, such as Sweden, Finland and Scotland (Babor et al., 2010; The Scottish Government, 2009), the withdrawal model may play out differently, but it remains likely that the influence of commercial arguments would increase as the justification for restrictive measures declines and other areas of public concern and policy claim attention and resources (Hawkins & McCambridge, 2020).

The primary consequences of the withdrawal model may be a relaxation of formal and informal controls on alcohol use and an increased risk of perpetuating Skog's ‘long waves’ of alcohol consumption, whereby consumption levels rise and fall in trends lasting several decades (Skog, 1986). Further consequences may include global corporations lobbying for the introduction of partnership arrangements in new jurisdictions, citing their apparent success in reducing youth drinking, and public health actors giving greater attention to emerging problems in low and middle income countries, where multinational corporations are seeking to expand their markets and secure influence over government policy-making (Babor et al., 2015). Alternatively, public health actors may focus on the alcohol problems they judge to be tractable in the context of limited interest from policy-makers who see bigger public health challenges to tackle around infectious disease control and societal inequalities. The alcohol problems public health actors focus on instead may include those with a high profile but narrow scope, such as recent UK campaigns on ‘children of alcoholics’, which were driven by the personal experiences of a small group of parliamentarians and prompted only minor policy shifts (BBC News, 2018a), albeit relating to high-risk and vulnerable groups. They may also include larger-scale campaigns that have uncertain benefits but are arguably less contentious than the WHO best-buy alcohol policy areas (i.e. pricing, availability and marketing) (World Health Organization, 2017). Examples of this may include recent campaigns around temporary abstinence (e.g. Dry January), calorie labelling and no- or low-alcohol drinks. Such a revised focus may be characterised in a less negative way as public health actors seeking incremental change (Cairney, 2012), rather than the radical change envisaged by the reinforcement model or the backward steps of the withdrawal model. This reflects the illustrative nature of our two models, which aim to alert stakeholders to potential pathways, and encourage debate around these, rather than make predictions.

## Conclusions

The decline in youth drinking is well-established and there is little sign that it is reversing, even if the largest changes may have already taken place. Young people also appear to be carrying their light consumption into adulthood in at least some jurisdictions. This is likely to deliver large public health benefits in the short-term and further benefits in the long-term, even if some of these are obscured in population-level data by the harms arising from heavier drinking generations. However, the impact of the decline in youth drinking on public policy and debate is less clear. We outline a reinforcement model where alcohol control policies ramp up following a cycle of apparent policy effectiveness, downward trends in drinking and harms and increased public support. We also outline a withdrawal model, which we regard as more likely. In this case, formal and informal controls on alcohol consumption erode over time as policy-makers address other challenges, public health actors struggle to make the case for continued intervention and industry actors reassert or strengthen their partnerships with governments around alcohol policy. These illustrative models indicate the challenges presented for public health actors by an improving public health trend (Whitaker et al., 2020).

Our analysis is necessarily speculative but has two key limitations. First, the authors are experienced in UK and Australian policy debate and this inevitably shapes our views. Those working in other policy contexts may see additional or alternative implications and we welcome debate on such points. Second, we focus on high-income countries but youth drinking is rising in many low- and middle-income countries (World Health Organization, 2014). As such, our analysis may conflict with a global perspective where the rising burden of alcohol-attributable harm in low and middle-income countries counteracts many, or all, of the health gains discussed above (Griswold et al., 2018).

Nonetheless, we have four tentative suggestions for public health actors. First, efforts should continue to understand why youth drinking is declining. This will support understanding of whether the decline will persist as today's young people move through adulthood and whether any policy measures can be reinforced to support the decline. To date, researchers have struggled to identify clear explanations for reduced youth drinking, despite many hypotheses. It appears increasingly likely that attention should focus on how a complex network of national and international influences is shaping the decline in different ways across the affected countries.

Second, public health actors should seek to understand children's, young people's and emerging adults’ attitudes towards alcohol and alcohol policy as well as the changing potency of key ideas within public debate (e.g. the vulnerable, unruly or deficient young person and the social and economic burden of chronic disease among older adults). This understanding is likely to be beneficial in developing future advocacy and intervention strategies that effectively harness changes in the policy context.

Third, public health actors may wish to continue advocating for governments to address the significant weaknesses in alcohol policy environments that exist in many countries, irrespective of consumption trends. These weaknesses include poorly structured tax systems, dysfunctional self-regulation of alcohol advertising by industry bodies and grossly inadequate provision of specialist alcohol treatment services (Alcohol Concern & Alcohol Research UK, 2018; Brennan et al., 2016; Sornpaisarn et al., 2015). Other advocacy messages may require adjustment however, such as the claim that there is a robust causal relationship between average levels of alcohol consumption within a population and the rate of alcohol-related harm (Room & Livingston, 2017). This claim is often made when arguing for population-level interventions affecting alcohol prices and availability but, as seen in the UK, the claim can be disproved when a sharp decline in drinking among young people reduces average consumption levels in the population while heavier drinking among older age groups drives up rates of alcohol-related harm (Holmes et al., 2022).

A final consideration returns to the analogy of the tobacco experience. This is instructive as it raises the question of whether the reinforcement model of evermore-restrictive alcohol policy is not only feasible but also desirable. Whereas the goal of a tobacco free world, or some close analogue, is largely uncontested within public health debate (Beaglehole et al., 2015), the ultimate goal of alcohol policy is less clear. The continuation of recent trends and the movement of generations of lighter drinking youth through the life course may prompt a reshaping of alcohol policy debates as harms from alcohol decline. This raises questions about the ultimate aims or end goals of alcohol policy that may need to be more rigorously considered by public health actors in coming decades.

## Funding sources

This work was supported by the 10.13039/100010269Wellcome Trust (208090/Z/17/Z) and an ARC Discovery Early Career Research Award to AP (DE190101074).

## Ethics approval

The authors declare that the work reported herein did not require ethics approval because it did not involve animal or human participation.

## Declarations of Interest

The authors declare that they have no known competing financial interests or personal relationships that could have appeared to influence the work reported in this paper.
